# Factor VIIa Regulates the Level of Cell-Surface Tissue Factor through Separate but Cooperative Mechanisms

**DOI:** 10.3390/cancers13153718

**Published:** 2021-07-23

**Authors:** Yahya Madkhali, Araci M. R. Rondon, Sophie Featherby, Anthony Maraveyas, John Greenman, Camille Ettelaie

**Affiliations:** 1 Biomedical Section, University of Hull, Cottingham Road, Hull HU6 7RX, UK; yammm95@hotmail.com (Y.M.); S.Featherby-2016@hull.ac.uk (S.F.); j.greenman@hull.ac.uk (J.G.); 2 Department of Medical Laboratories, College of Applied Medical Sciences, Majmaah University, P.O. Box 66, Majmaah 11952, Saudi Arabia; 3 Einthoven Laboratory for Vascular and Regenerative Medicine, Department of Internal Medicine, Division of Thrombosis and Hemostasis, Leiden University Medical Center, 2333 ZA Leiden, The Netherlands; A.M.da_Rocha_Rondon@lumc.nl; 4 Division of Cancer-Hull York Medical School, University of Hull, Cottingham Road, Hull HU6 7RX, UK; anthony.maraveyas@hey.nhs.uk

**Keywords:** tissue factor, factor VIIa, protease-activated receptor-2, caveolae, cholesterol-rich microdomins, cell signalling

## Abstract

**Simple Summary:**

Under normal conditions, blood coagulation is suppressed to prevent thrombosis. However, during inflammatory conditions such as injury or disease conditions, the protein “tissue factor (TF)” is expressed on the surface of the cells and is also released into the bloodstream within cell-derived vesicles called “microvesicles”. TF appears first at the site of trauma which makes TF suitable for determining the extent of damage and instructing cells to proliferate and repair, or if severely damaged, to die. The relationship between cancer and thrombosis was reported in the early part of the 19th century. Cancer cells and particularly those with aggressive tendencies have the ability to produce, and then optimise the amount of TF on the cell, in order to maximise the pro-survival and proliferative properties of this protein. This study has demonstrated some of the mechanisms by which cells control excessive amounts of TF, to levels ideal for tumour survival and growth.

**Abstract:**

Procoagulant activity of tissue factor (TF) in response to injury or inflammation is accompanied with cellular signals which determine the fate of cells. However, to prevent excessive signalling, TF is rapidly dissipated through release into microvesicles, and/or endocytosis. To elucidate the mechanism by which TF signalling may become moderated on the surface of cells, the associations of TF, fVII/fVIIa, PAR2 and caveolin-1 on MDA-MB-231, BxPC-3 and 786-O cells were examined and compared to that in cells lacking either fVII/fVIIa or TF. Furthermore, the localisation of labelled-recombinant TF with cholesterol-rich lipid rafts was explored on the surface of primary human blood dermal endothelial cells (HDBEC). Finally, by disrupting the caveolae on the surface of HDBEC, the outcome on TF-mediated signalling was examined. The association between TF and PAR2 was found to be dependent on the presence of fVIIa. Interestingly, the presence of TF was not pre-requisite for the association between fVII/fVIIa and PAR2 but was significantly enhanced by TF, which was also essential for the proliferative signal. Supplementation of HDBEC with exogenous TF resulted in early release of fVII/fVIIa from caveolae, followed by re-sequestration of TF-fVIIa. Addition of labelled-TF resulted in the accumulation within caveolin-1-containing cholesterol-rich regions and was also accompanied with the increased assimilation of cell-surface fVIIa. Disruption of the caveolae/rafts in HDBEC using MβCD enhanced the TF-mediated cellular signalling. Our data supports a hypothesis that cells respond to the exposure to TF by moderating the signalling activities as well as the procoagulant activity of TF, through incorporation into the caveolae/lipid rafts.

## 1. Introduction

The release of a factor, derived from injured tissue and capable of inducing blood coagulation, was recorded as early as 1834 [1,2]. This was later termed “thromboplastin” or “thombokinase” [3], and more recently as tissue factor (TF). The resulting formation of TF-fVIIa complex and its role in the initiation of the coagulation system has been reported for over four decades [4,5,6,7,8]. More recently, TF-fVIIa complex on cell surfaces was shown to regulate a number of cellular functions including cell proliferation and survival [9,10,11]. Moreover, our recent studies have shown that the accumulation of TF in endothelial cells can induce apoptosis [12,13,14]. Both proliferative and pro-apoptotic signalling arising from TF are initiated by, and require, the activation of PAR2 [15,16]. The formation of TF-fVIIa or TF-fVIIa-fXa complexes can promote PAR2 activation [17,18]. Blocking of the formation of TF-fVIIa complex using HTF-1 antibody, inhibition of fVIIa proteolytic activity using a polyclonal inhibitory antibody, or the prevention of PAR2 activation using SAM11 antibody, all suppressed TF-mediated alterations in cell proliferation and apoptosis [15,16].

The activation of PAR2 also promotes the release of procoagulant MV which carries TF from the cell surface [19]. This release of TF contributes to a mechanism by which the cells can moderate and manage the excessive amounts of TF on the surface of the cell. We previously demonstrated that repeated treatment of endothelial cells with recombinant TF reduced the amount of fVII/fVIIa stores available within cells, the magnitude of which correlated with TF released within MV [15]. The repetitive exposure of cells to TF and the depletion of cellular fVII/fVIIa reserves resulted in impairment of PAR2 activation and TF release within MV. However, studies have also shown that TF may be taken up by cells through endocytotic processes and may also be recycled [20,21,22,23]. The internalisation of TF-fVIIa complex has been reported to be mediated through both a clathrin-dependent mechanism [21] and clathrin-independent processes [22]. Furthermore, TF can localise with cholesterol-rich membrane micro-domains on the surface of cells, including the caveolae and lipid rafts [24,25,26,27,28,29]. Caveolae have been suggested to dynamically harbour and regulate the procoagulant activity of TF [25]. In fact, various studies have suggested that the TF protein, either on its own, or in complex with fVIIa, can associate with caveolae and lipid rafts [24,25,26,27,28,29]. Depletion of cholesterol from the cell membrane significantly reduced the amount of TF associated with lipid rafts [24] but also resulted in increased TF procoagulant activity on the cell surface [30]. Therefore, the caveolae-associated TF may act as a latent pool which may become activated, resulting in the release of the caveolae content. This may also act as a repository to regulate TF activity. However, the role of endogenous fVII/fVIIa in the regulation of these events and the subsequent contribution to the regulation of TF signalling is largely unexplored. In this study, the associations between TF, fVII/fVIIa, PAR2 and caveolin-1 were examined and the contributions of TF and fVIIa to these associations investigated. Proximity ligation assay (PLA) has been used by our group and other investigators [29,31,32] to successfully study TF interactions. This procedure, together with studies on co-localisation with cholesterol-rich micro-domains, enabled the analysis of different sets of cell-surface proteins, in situ.

## 2. Material and Methods

### 2.1. Cell Culture and Analysis of Cell Numbers

Human dermal blood primary endothelial cells (HDBEC), devoid of endogenous TF, were cultured in MV media containing 5% (*v*/*v*) foetal calf serum (FCS) and growth supplements (PromoCell, Heidelberg, Germany). Cell lines were obtained from ATCC-LGC (Teddington, UK). MDA-MB-231 breast cancer cells were cultured in DMEM supplemented with 10% (*v*/*v*) FCS. BxPC-3 pancreatic cancer cells and 786-O kidney cancer cells (ATCC, Teddington, UK) were cultured in RPMI-1640 supplemented with 10% (*v*/*v*) FCS. MDA-MB-231 TF-KO cells were a gift and were cultured similarly to the wild-type cells [33]. The lack of TF on the surface of MDA-MB-231 TF-KO cells was confirmed beforehand by flow cytometry. Cell numbers were determined by crystal violet staining using a kit obtained from Active Motif (La Hulpe, Belgium) as previously described and confirmed [13,34]. Cell numbers were interpreted from standard curves, constructed separately for each cell type.

### 2.2. Suppression of fVII/fVIIa Expression by siRNA Transfection

A set of “Silencer-Select” pre-designed siRNA (Life Technologies, Paisley, UK) specific for the coagulation factor VII was used to suppress the expression of fVII/fVIIa protein in cell lines. The knock-down was optimised beforehand by measuring fVIIa antigen and activity over a range of 0–200 nM. Transfections were carried out using Trans IT-2020 transfection reagent (Mirus Bio LLC, Madison, WI, USA) as described before [15,16] and the cells were incubated for 48 h at 37 °C. To confirm the suppression of fVII/fVIIa expression, samples of cells were examined by Western blot using a rabbit polyclonal anti-fVII antibody 1:2000 (*v*/*v*; Abcam, Cambridge, UK) which was then developed with a goat anti-rabbit antibody 1:4000 (*v*/*v*; Santa Cruz Biotechnologies, Heidelberg, Germany). The expression levels were normalised against the respective GAPDH (Santa Cruz Biotechnologies) in each sample. The blots were analysed using the ImageJ program and the level of expression of fVII as well as the active subunits of fVIIa protein assessed.

### 2.3. Duolink Proximity Ligation (PLA) Assay

All procedures were carried out using the Duolink reagents (Sigma Chemical Company Ltd., Poole, UK) and adapted from that previously described in detail [31,32]. All cells (10^3^), were seeded out into 35 mm-glass based μ-dishes (InVitro Scientific/Cellvis, Sunnyvale, CA, USA) in respective media overnight. In some experiments, the cells were treated with recombinant TF (Innovin recombinant human tissue factor reagent; Dade Behring, Liederbach, Germany) according to the TF activity (1 U/mL = 1.3 ng/mL). In other experiments, cells were treated with fVIIa (Enzyme Research Laboratories Ltd., Swansea, UK) or transfected with siRNA, as stated in the Results section. Cells were then fixed with 4% (*v*/*v*) paraformaldehyde for 15 min and washed three times with PBS. All samples were blocked with Duolink blocking buffer for 1 h and incubated overnight at 4 °C, with combinations of antibodies as follows. To examine the interaction between fVII/fVIIa and TF, cells were probed with a mouse anti-fVII antibody (321621; 10 µg/mL; R&D Systems, Abingdon, UK) together with a rabbit anti-TF antibody (FL295; 5 µg/mL; Santa Cruz Biotechnologies). When testing the proximity between TF and PAR2, the cells were probed with a rabbit anti-TF antibody (FL295; 5 µg/mL) together with mouse anti-PAR2 antibody (SAM11; 20 μg/mL; Bio-Rad, Hemel Hempstead, UK). In addition, sets of cells were probed with a polyclonal rabbit anti-fVII antibody (10 µg/mL) and a mouse anti-PAR2 antibody (SAM11; 20 μg/mL) to examine the association of fVII/fVIIa and PAR2 proteins. In addition, the association of caveolin-1 with either TF or fVII/fVIIa was examined using a polyclonal rabbit anti-caveolin-1 antibody (10 μg/mL; GenTex, Irvine, CA, USA) together with a mouse anti-TF antibody (10H10; 5 µg/mL; Bio-Rad) or a mouse anti-fVII antibody (321621; 10 µg/mL), respectively. To ensure specificity, the secondary antibodies (probes) were omitted and the assay carried out to ensure specificity [32]. In addition, throughout the various experiments, some of the primary antibodies were substituted in turn, either with rabbit or mouse IgG isotypes as appropriate (New England Biolabs, Hitchin, UK; 20 μg/mL and 10 µg/mL, respectively). Following the incubations, the cells were washed and PLA performed as described by the manufacturer. The cells were then stained with DAPI (2 μg/mL) and in certain experiments, also with Phalloidin-FITC (2 µg/mL). Images were acquired using a Zeiss Axio Vert.A1 inverted fluorescence microscope with a × 40 magnification (Carl Zeiss Ltd., Welwyn Garden City, UK). The number of red fluorescent events and the number of nuclei were determined from 10 fields of view for each assay using the ImageJ program.

### 2.4. Disruption and Labelling of Caveolae by MβCD and NBD-Cholesterol Exchange

Disruption of caveolae was achieved by cholesterol depletion using methyl-β cyclodextrin (MβCD; Sigma Chemical Co Ltd.) according to the procedures described [35] and previously optimised in our laboratory [31]. HDBEC (2 × 10^4^) were seeded out in 96-well plates containing complete medium and incubated overnight. These cells were used since they do not express endogenous TF under normal culture conditions. On the following day, the media was removed and the cells were treated with a range of concentrations of MβCD (0–5 mM) diluted in serum-free MV medium for 1 h at 37 °C. After the incubation, the cells were washed twice with PBS and incubated with complete MV medium, in the presence or absence of TF (4 U/mL = 5.2 ng/mL) for 24 h at 37 °C. To determine the optimal concentration of MβCD and to maintain cell viability, cell numbers were then determined using a crystal violet assay as above. Cellular apoptosis was quantified using the TiterTACS™ Colorimetric Apoptosis Detection Kit (AMS Biotechnology, Abingdon, UK) according to the manufacturer’s instructions [13,15].

The membrane-associated cholesterol was also partially replaced with 3-dodecanoyl-NBD cholesterol (Cayman Chemicals/Cambridge Bioscience Ltd., Cambridge, UK) using MβCD, in order to visualise lipid rafts by fluorescence microscopy (Ext. 465 nm, Em. 535 nm). HDBEC (10^3^) were seeded into 35 mm glass bottom dishes and permitted to adhere. Labelling was carried out by incubation with the various MβCD:NDB-cholesterol complex and incubated for 1 h at 37 °C. Initially, the ratio of MβCD:NBD-cholesterol was optimised using molar ratios of 6:1, 8:1 and 12:1. In some experiments, the cells were also supplemented with the Texas Red-labelled recombinant TF. The media was then discarded, and the cells were fixed, washed with PBS, and stained with DAPI (2 μg/mL) for 10 min. The cells were finally washed twice with PBS and visualized as described below.

### 2.5. Preparation of Texas Red-Labelled TF

Human recombinant TF (1 μg/mL) was labelled with a Texas Red tag using the Innova Lightning-link Texas Red kit (Expedeon, Cambridge, UK) according to the manufacturer’s instructions. Briefly, TF (1 μg/mL) was diluted with the LL-Modifier reagent provided with the kit at a ratio 10:1 (*v*/*v*). The mixture was then added into the lyophilized Lightning-Link^®^ vial, gently shaken and then incubated overnight at room temperature in the dark. The LLquencher reagent provided with the kit was then added to the vial at a ratio 1:4 (*v*/*v*) and incubated at room temperature for 30 min in the dark to neutralise any unbound Texas Red. Prior to use, TF activity was established by an fXa-generation assay and TF labelling confirmed by capture using an anti-TF antibody-coated plate and measuring the fluorescence. HDBEC were seeded out into 35 mm glass bottom dishes and supplemented with the labelled Texas Red-TF (4 U/mL = 5.2 ng/mL) and incubated for up to 40 min. The cells were then fixed and stained with DAPI (2 μg/mL) and were analysed by confocal microscopy at room temperature using a Zeiss LSM 710 confocal microscope (with a ×63 water immersion objective), and images were acquired using the ZEN software (Carl Zeiss Ltd.). To measure TF activity, the samples were incubated with fVIIa (10 nM; Enzyme Research Labs, Swansea, UK) in Tris-HCl-saline buffer pH 7.4, containing 5 mM CaCl_2_, together with fX (100 nM) and the fXa substrate (0.2 mM; Hyphen BioMed/Quadratech, Epsom, UK) diluted in the same buffer (200 µL). The samples were incubated for 20 min to develop the colour and then quenched using 2% (*v*/*v*) acetic acid (50 µL) and the absorptions measured immediately at 410 nm. The amount of fXa generated was determined using a standard curve prepared using fXa (0–20 nM; Enzyme Research Labs).

### 2.6. Statistical Analysis

All data represent the calculated mean values from the number of experiments stated in each figure legend ± the calculated standard error of the mean. Statistical analysis was carried out using the Statistical Package for the Social Sciences (SPSS Inc. Chicago, IL, USA), and significance was determined by a paired *t*-test.

## 3. Results

The specificity of the proximity ligation assay (PLA) was demonstrated by substituting one of the primary antibodies with an isotype antibody. Representative of these control samples, encompassing all the antigens tested for, are presented in the following figures, each depicting a different antibody that was replaced.

### 3.1. Assessment of the Interaction of TF, fVII/fVIIa and PAR2 on the Surface of Cells

**Figure 1:**
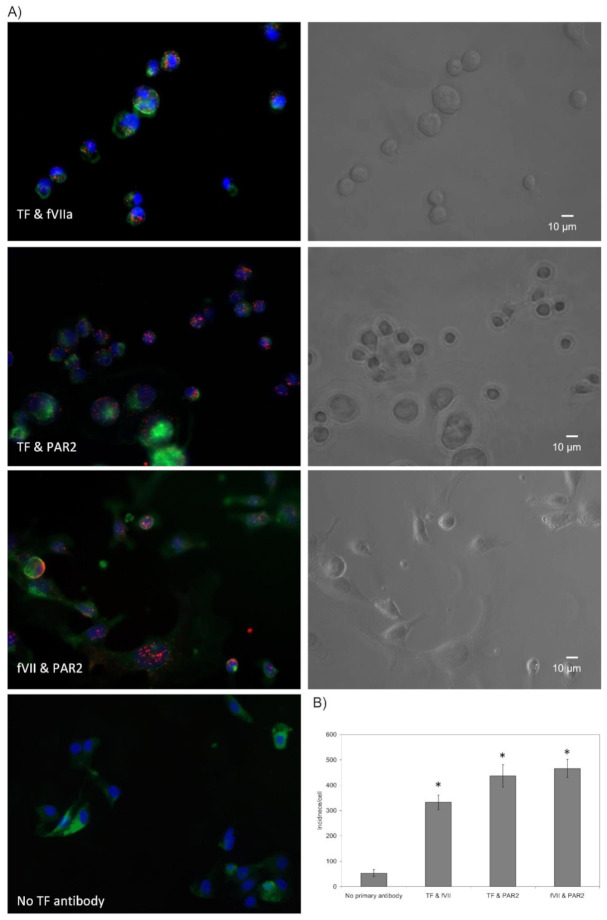
Analysis of the association between TF, fVII and PAR2 on the surface of cells: MDA-MB-231 cells (10^3^) were seeded out into 35 mm-glass based μ-dishes and incubated overnight. Cells were then fixed with 4% (*v*/*v*) paraformaldehyde for 15 min, washed three times with PBS for 5 min and blocked with Duolink blocking buffer for 1 h. The cells were then incubated overnight at 4 °C with combinations of antibodies as follows: the cells were probed with a mouse anti-fVII antibody (321621; 10 µg/mL) together with a rabbit anti-TF antibody (FL295; 5 µg/mL) to examine the interaction between fVII and TF. Another set of cells were probed with a rabbit anti-TF antibody (FL295; 5 µg/mL) and a mouse anti-PAR2 antibody (SAM11; 20 μg/mL) to examine the association of TF and PAR2 proteins. Finally, to test the proximity between fVII and PAR2, the cells were probed with a polyclonal rabbit anti-fVII antibody (10 µg/mL) together with mouse anti-PAR2 antibody (SAM11). (**A**) The dishes were washed three times with PBS and PLA performed according to the manufacturer’s instructions. The cells were stained with DAPI (2 μg/mL) and Phalloidin-FITC (2 µg/mL). Images were acquired using a Zeiss Axio Vert.A1 inverted fluorescence microscope with a ×40 magnification. A different representative of the control samples in which one primary antibody was substituted with a isotype control is presented in each of the figures. (**B**) The number of red fluorescent events and nuclei were determined as incidence/cell in 10 fields of view from each assay using the ImageJ program. (*n* = 4; * = *p* < 0.05 vs. the control).

**Figure 2:**
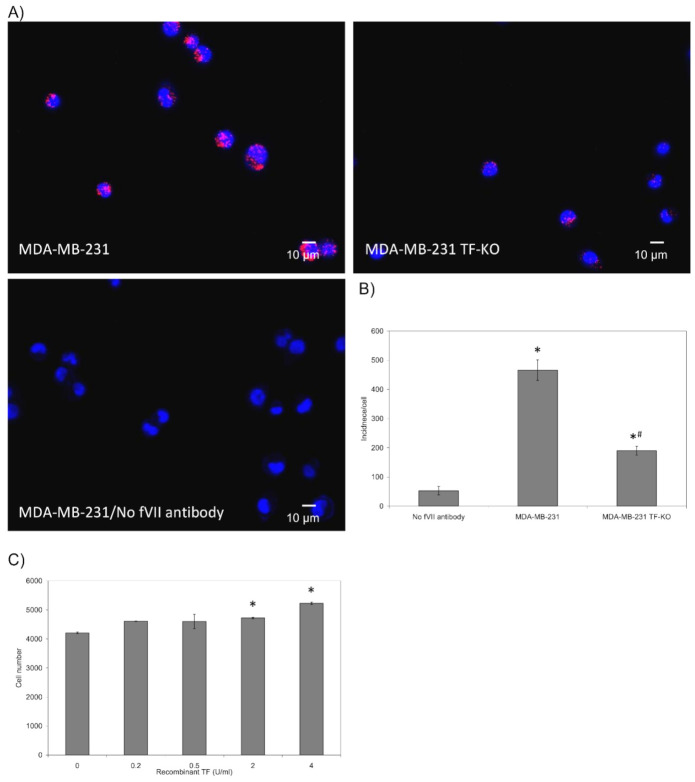
Analysis of the association of fVII and PAR2 in the presence and absence of TF: Wild-type MDA-MB-231 and MDA-MB-231 TF-KO cells (10^3^) were seeded out into 35 mm-glass based μ-dishes overnight. Cells were then fixed with 4% (*v*/*v*) paraformaldehyde for 15 min, washed three times with PBS for 5 min and blocked with Duolink blocking buffer for 1 h. The cells were then incubated overnight at 4 °C with a polyclonal rabbit anti-fVII antibody (10 µg/mL) and a mouse anti-PAR2 antibody (SAM11; 20 μg/mL). (**A**) The dishes were washed three times with PBS and PLA performed according to the manufacturer’s instructions. The cells were stained with DAPI (2 μg/mL). Images were acquired using a Zeiss Axio Vert.A1 inverted fluorescence microscope with a ×40 magnification. A different representative of the control samples in which one primary antibody was substituted with a isotype control is presented in each of the figures. (**B**) The number of red fluorescent events and nuclei were determined as incidence/cell in 10 fields of view from each assay using the ImageJ program. (*n* = 4; * = *p* < 0.05 vs. the control; # = *p* < 0.05 vs. Wild type MDA-MB-231 cells). (**C**) MDA-MB-231 TF-KO cells (2 × 10^3^) cells were seeded out in 24 well plated and supplemented with a range of recombinant TF (0–4 U/mL). Cell numbers were determined at 48 h, by crystal violet staining. (*n* = 3; * = *p* < 0.05 vs. No TF sample).

**Figure 3:**
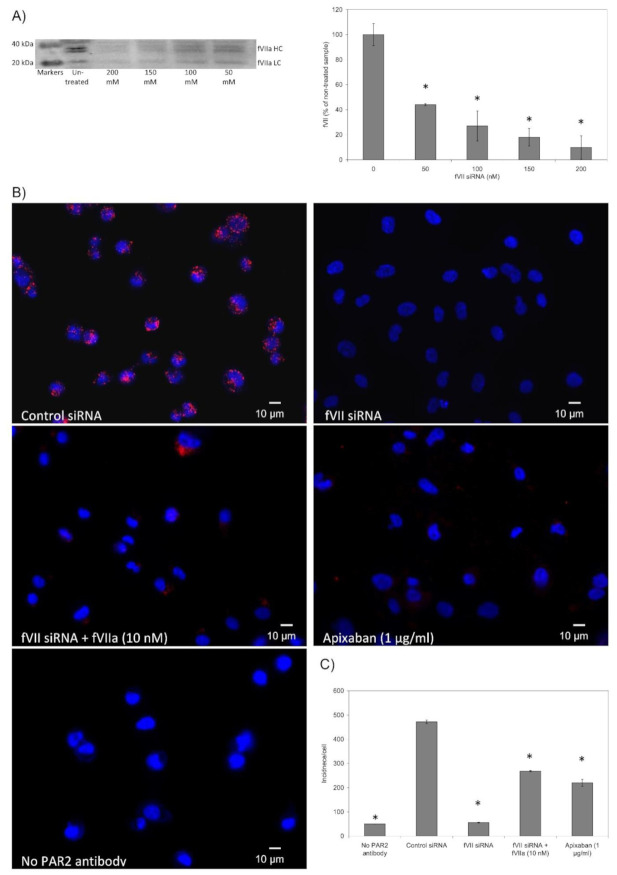
Analysis of the association of TF and PAR2 in the presence and absence of fVIIa: Sets of MDA-MB-231 cells (10^5^) cells were transfected with a set of Silencer Select Pre-designed siRNA specific for the coagulation factor VII or a control siRNA (200 nM) using Trans IT-2020 transfection reagent and incubated for 48 h at 37 °C. (**A**) The knock-down was optimised beforehand by Western blot using a rabbit polyclonal anti-fVII antibody 1:2000 (*v*/*v*) and developed with a goat anti-rabbit antibody 1:4000 (*v*/*v*) (also see Figure S4). The membranes were analysed by the ImageJ program to determine the level of expression of fVII as well as the active subunits of fVIIa protein. (*n* = 3; * = *p* < 0.05 vs. Non-transfected sample). (**B**) MDA-MB-231 cells (10^3^) were seeded out into 35 mm-glass based μ-dishes overnight. Sets of cells were transfected and incubated as above. Some transfected cells were supplemented with purified fVIIa (10 nM) and other untransfected sets of cells were treated with apixaban (1 µg/mL). All cells were then fixed with 4% (*v*/*v*) paraformaldehyde for 15 min, washed three times with PBS for 5 min and blocked with Duolink blocking buffer for 1 h. The cells were then incubated overnight at 4 °C with a rabbit anti-TF antibody (FL295; 5 µg/mL) and a mouse anti-PAR2 antibody (SAM11; 20 μg/mL). The dishes were washed three times with PBS and PLA performed according to the manufacturer’s instructions. The cells were stained with DAPI (2 μg/mL) and images were acquired using a Zeiss Axio Vert.A1 inverted fluorescence microscope with a ×40 magnification. A different representative of the control samples in which one primary antibody was substituted with a isotype control, is presented in each of the figures. (**C**) The number of red fluorescent events and nuclei were determined as incidence/cell in 10 fields of view from each assay using the ImageJ program. (*n* = 3; * = *p* < 0.05 vs. Sample containing control siRNA).

Analysis of the association of PAR2 with either TF or fVII/fVIIa on the surface of MDA-MB-231 cells indicated similar levels of associations (Figure 1A,B). Interestingly, the association between TF and fVII/fVIIa was lower. Similar analysis carried out in BxPC-3 and 786-O cells produced significantly lower associations between these three proteins (Supplementary Materials Figure S1), possibly due to lower expression of fVII/fVIIa in these two cells lines, compared to MDA-MB-231 cells [15]. The comparison of wild-type MDA-MB-231 with MDA-MB-231 TF-KO cells also indicated some association between fVII/fVIIa and PAR2 in both cells lines, although this was significantly lower in the absence of TF (Figure 2A,B). In order to further examine the functional significance of TF in proliferative signalling, MDA-MB-231 TF-KO cells were supplemented with a range of concentrations of recombinant TF (0–4 U/mL) and cell number were examined after 48 h. In support of the above data, supplementation of the MDA-MB-231 TF-KO cells promoted the proliferation of these cells (Figure 2C), although this was at a reduced rate compared to that established for the wild-type MDA-MB-231 cells [32]. Subsequently, siRNA-mediated knock-down of the fVII/fVIIa in all three cell lines was carried out as described before [15,16] and shown to be effective in limiting the expression of cellular fVII/fVIIa in MDA-MB-231 cells, to approximately 10% of the untreated cells (Figure 3A). Furthermore, in agreement with previous studies, the detected bands corresponded to the heavy and light chains of the active form of fVIIa (Figure 3A), while little zymogen fVII was previously detected in these cells [15,16,36]. Suppression of the expression of fVII/fVIIa in MDA-MB-231 cells resulted in a significant reduction in the association between TF and PAR2 (Figure 3B,C). Supplementation of these cells with purified fVIIa resulted in a modest recovery in the number of interactions but was localised to some of the cells. Suppression of the expression of fVII/fVIIa in BxPC-3 and 786-O cells line almost abolished any interaction between these proteins (Figure S2). Additionally, we previously reported the ability to apixaban to interfere with the proteolytic and signalling activity of fVIIa. Therefore, samples of MDA-MB-231 cells were pre-incubated with apixaban (1 µg/mL) for 30 min prior to analysis. The proximity between TF and PAR2 was shown to be significantly reduced on pre-incubation with apixaban suggesting a facilitating role for fVIIa, during complex formation between these two proteins.

### 3.2. In the Presence of fVIIa, Exogenous TF Translocates to the Caveloae on Endothelial Cells

**Figure 4:**
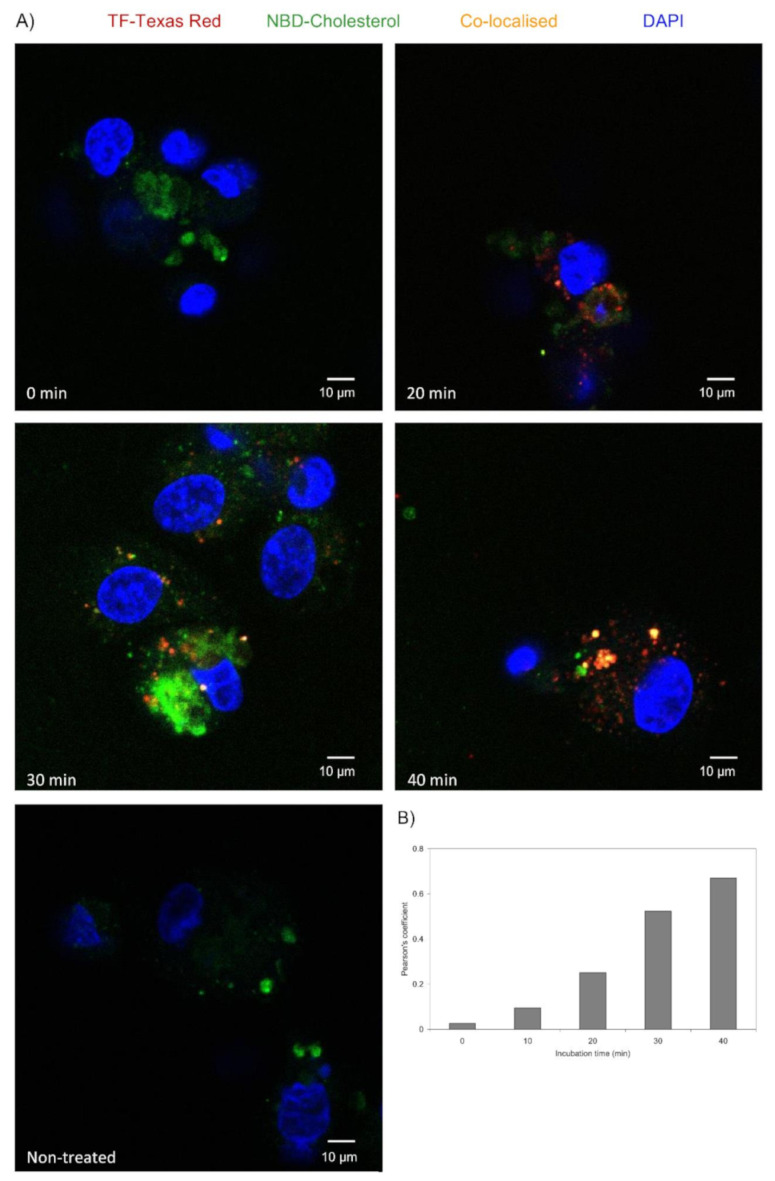
Time-course of the co-localisation of TF-Texas Red with NBD-labelled cholesterol on the cell surface: Human recombinant TF (1 μg/mL) was labelled with a Texas Red tag using the Innova Lightning-link Texas Red kit according to the manufacturer’s instructions. TF activity was established by fXa-generation assay and TF labelling confirmed before use. (**A**) HDBEC (10^3^) were seeded into 35 mm glass bottom dishes and labelled using NBD-cholesterol to highlight the cholesterol-rich lipid rafts. The cells were then supplemented with the labelled Texas Red-TF (5.2 ng/mL) and incubated for up to 40 min. The cells were then fixed and stained with DAPI (2 μg/mL) and were analysed by confocal microscopy at room temperature, using a Zeiss LSM 710 confocal microscope (with a ×63 water immersion objective) and images were acquired using the ZEN software. (**B**) For comparison, co-localisation overlap coefficient values were determined in 10 fields of view from each assay using the ImageJ program.

**Figure 5:**
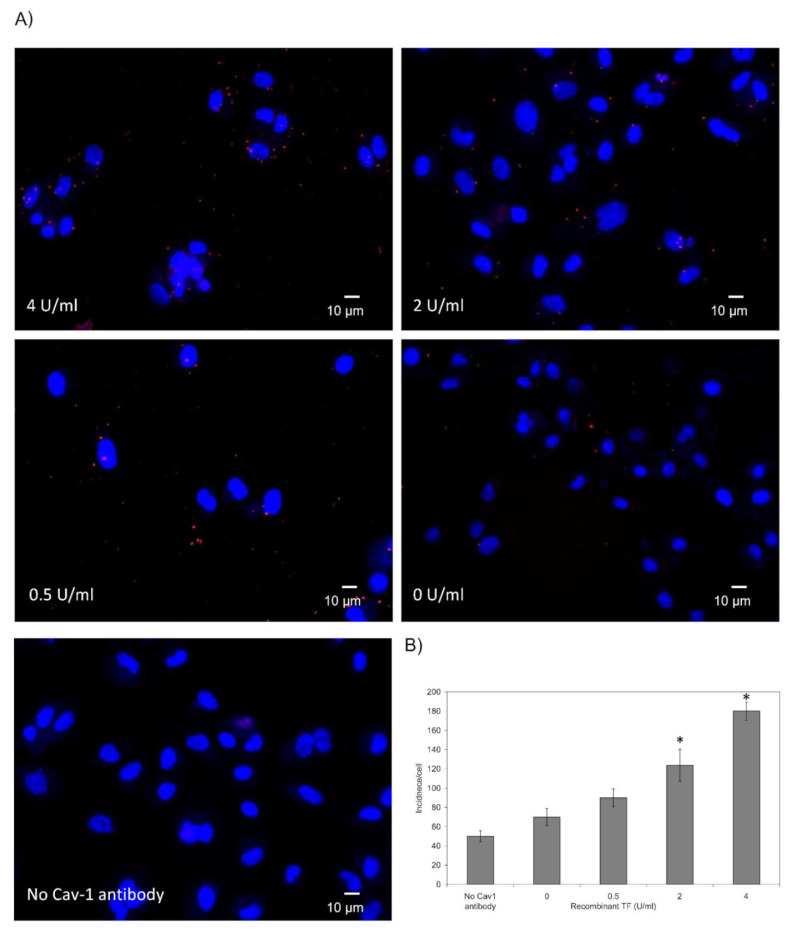
Analysis of the association of recombinant TF with caveolin-1 on the surface of HDBEC: HDBEC cells (2 × 10^3^) were seeded out into 96-well plate based μ-dishes overnight and treated with recombinant TF (4 U/mL). Cells were then fixed with 4% (*v*/*v*) paraformaldehyde for 15 min, washed three times with PBS for 5 min and blocked with Duolink blocking buffer for 1 h. The cells were then incubated overnight at 4 °C with a mouse anti-TF antibody (10H10; 5 µg/mL) and a polyclonal rabbit anti-caveolin-1 antibody (10 μg/mL). (**A**) The dishes were washed three times with PBS and PLA performed according to the manufacturer’s instructions. The cells were stained with DAPI (2 μg/mL) and images were acquired using a Zeiss Axio Vert.A1 inverted fluorescence microscope with a ×40 magnification. A different representative of the control samples in which one primary antibody was substituted with a isotype control, is presented in each of the figures. (**B**) The number of red fluorescent events and nuclei were determined as incidence/cell in 10 fields of view from each assay using the ImageJ program. (*n* = 3; * = *p* < 0.05 vs. No TF sample).

**Figure 6:**
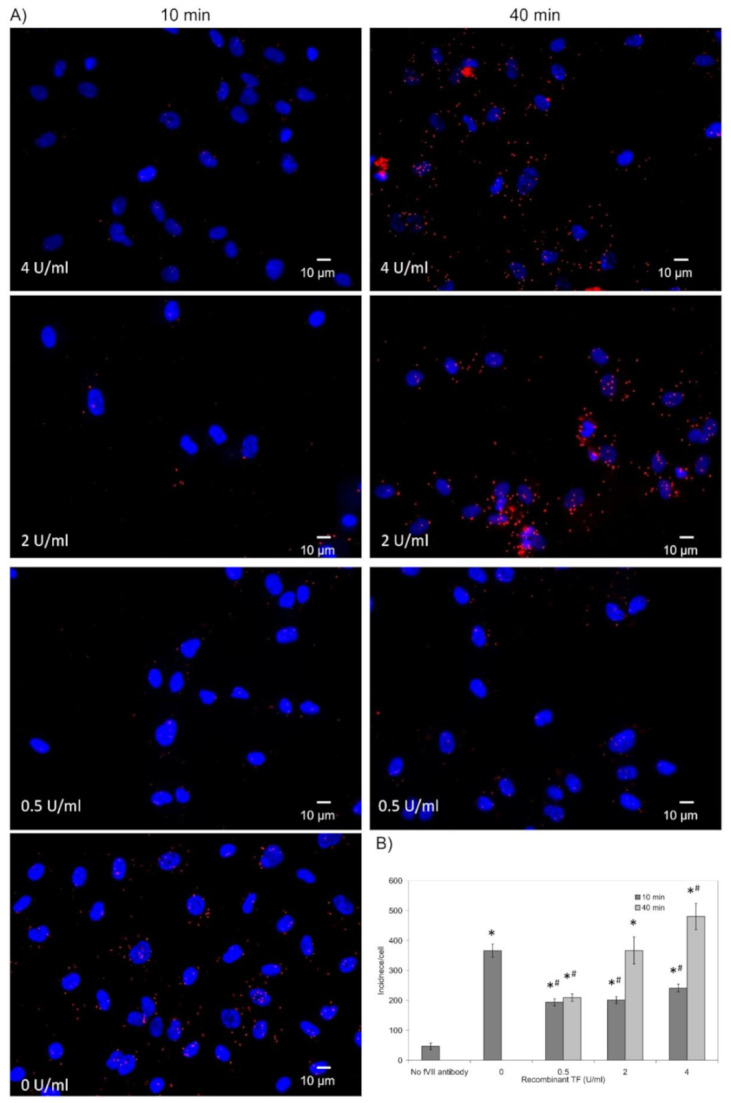
Analysis of the association of recombinant fVIIa with caveolin-1 on the surface of HDBEC on addition of recombinant TF: HDBEC (10^3^). were seeded out into 35 mm-glass based μ-dishes overnight and treated with recombinant TF (1–4 U/mL) for 10 or 40 min. Cells were then fixed with 4% (*v*/*v*) paraformaldehyde for 15 min, washed three times with PBS for 5 min and blocked with Duolink blocking buffer for 1 h. The cells were then incubated overnight at 4 °C with a mouse anti-fVII antibody (321621; 10 µg/mL) and a polyclonal rabbit anti-caveolin-1 antibody (10 μg/mL). (**A**) The dishes were washed three times with PBS and PLA performed according to the manufacturer’s instructions. The cells were stained with DAPI (2 μg/mL) and images were acquired using a Zeiss Axio Vert.A1 inverted fluorescence microscope with a ×40 magnification. (**B**) The number of red fluorescent events and nuclei were determined as incidence/cell in 10 fields of view from each assay using the ImageJ program. (*n* = 3; * = *p* < 0.05 vs. No fVII antibody; # = *p* < 0.05 vs. No TF sample).

HDBEC were employed to explore the localisation of TF and fVII/fVIIa within the caveolae, in response to exogenous TF because these cells are known to be devoid of endogenous TF expression under normal culture conditions [19]. In order to visualise the lipid rafts/caveolae, HDBEC were enriched with NBD-cholesterol, and the exchange was optimised using a range molar ratios (Figure S3). A preliminary study of the cells at 24 h did not indicate any deleterious outcome on cell numbers at lower concentrations of MβCD (see later figures showing cell numbers). Incubation of labelled HDBEC with Texas Red-labelled recombinant TF indicated the co-localisation of TF with cholesterol-rich lipid rafts by 40 min but was observable as early as 20 min (Figure 4A,B). Consequently, analysis of the association of recombinant TF with caveolin-1 by PLA indicated a TF dose-dependent increase in the association between these proteins on the surface of HDBEC, after 40 min incubation (Figure 5A,B). Moreover, examination of the association between fVII/fVIIa and caveolin-1 by PLA, following treatment with recombinant TF indicated an initial reduction in fVII/fVIIa-caveolin-1 association which then recovered to levels even higher than those observed for the untreated sample. However, this recovery was dependent on the dose of recombinant TF added and was absent with 0.5 U/mL TF (Figure 6A,B).

### 3.3. Lipid Rafts Moderate the Signalling Function of TF-FVIIa on Endothelial Cells

**Figure 7:**
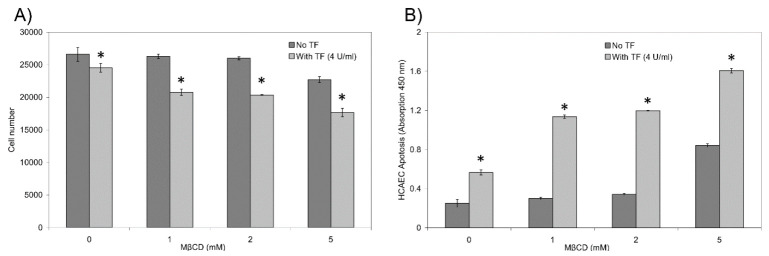
Analysis of the influence of recombinant TF on cell viability before and after cholesterol depletion: HDBEC (2 × 10^4^) were seeded in 96-well plates containing complete medium and incubated overnight. On the following day, the media was removed, and the cells were treated with a range of concentrations of MβCD (0–5 mM) diluted in serum-free MV medium (*w*/*v*) for 1 h at 37 °C. After the incubation, the cells were washed twice with PBS and incubated with complete MV medium, in the presence or absence of TF (4 U/mL = 5.2 ng/mL) for 24 h at 37 °C. (**A**) Cell numbers were then determined using crystal violet assay as above. (*n* = 4; * = *p* < 0.05 vs. the respective sample without MβCD). (**B**) Cellular apoptosis was quantified in selected samples using the TiterTACS™ Colorimetric Apoptosis Detection Kit (AMS Biotechnology, Abingdon, UK) according to the manufacturer’s instructions. (*n* = 3; * = *p* < 0.05 vs. the respective sample without TF).

We have previously shown that disruption of cholesterol-rich micro-domains prevents protease-activated receptor 2 (PAR2)-mediated alterations in cell surface tissue factor activity [31]. In this study, we also examined the hypothesis that the pro-apoptotic signal exerted by the exposure of cells to TF may also be moderated by these domains. Incubation with a high concentration of recombinant TF (4 U/mL) is known to induce cellular apoptosis in HDBEC [15], a response that is dissimilar to that observed in MDA-MB-231 cells. Therefore, while supplementation of otherwise untreated HDBEC with recombinant TF resulted in the reduction in viable cells after 24 h, the disruption of cholesterol-rich micro-domains using MβCD significantly enhanced the pro-apoptotic function of recombinant TF (Figure 7).

## 4. Discussion

The disruption of cellular layers occurs as a consequence of injury and leads to the exposure of TF and the activation of coagulation at the site of injury, in order to arrest bleeding. Moreover, TF appears to instruct cells to divide or become apoptotic through mechanisms that are initiated on the cell surface. In response to TF, cells attempt to moderate the amount of active TF on the cell membrane by incorporation and release into MV, as well as by endocytosis. In this study, Proximity Ligation Assay was used to demonstrate the association between TF and PAR2, fVIIa and PAR2, as well as between TF and fVIIa suggesting the formation of a ternary complex (Figure 1). The lower level of association observed between TF and fVIIa, in comparison to other measurements, suggests that the complex formation between PAR2 and either of these proteins is not contingent on conditions that are essential for the initiation of coagulation. PAR2 is activated by the proteolytic action of fVIIa in complex with TF [17,18] and leads to cell proliferation in MDA-MB-231 cells. Therefore, MDA-MB-231 TF-KO cells were used to examine the association between fVII/fVIIa and PAR2 in the absence of TF. Analysis of the association of fVII/fVIIa with PAR2 in MDA-MB-231 TF-KO cells showed a 60% reduction compared to that in the wild-type cell (Figure 2). Therefore, in the absence of TF, fVII/fVIIa may associate with PAR2 but does not appear to be effective in activating PAR2 protein. This suggests a feasible mechanism by which the cells gauge the amount of TF they come into contact with, or that is expressed on the surface of cells, and therefore can regulate how much TF is released without excessive MV production. In contrast, fVIIa appears to be pre-requisite to complex formation between TF and PAR2, as well as the activation of the latter protein (Figure 3). This was also evident by the lower interactions between TF and PAR2, observed in BxPC-3 and 786-O cells which express TF and PAR2 but low levels of fVIIa [15]. The reduction in TF-PAR2 proximity was also observed on pre-incubation of the MDA-MB-231 cells with apixaban. We previously showed the ability of this molecule to inhibit both the proteolytic and signalling activity of fVIIa [16]. Therefore, it is proposed that the catalytic domain of fVIIa is essential for the approximation of TF and PAR2 and the formation of the tertiary complex TF-fVIIa-PAR2.

Depending on the magnitude of inflammation, the presence of excessive amounts of TF may overwhelm the efficient clearance through MV release. Therefore, an alternative cellular mechanism involving caveolae-mediated endocytosis could at least temporarily moderate the excessive amounts of TF present on the cell surface. In fact, it has been shown that caveolae are capable of harbouring TF [27,28]. To examine this hypothesis, the association of TF with caveolae was examined in HDBEC. Following supplementation of HDBEC with Texas Red conjugated TF, increasing amounts co-localised within cholesterol-rich domains/lipid rafts within 40 min (Figure 4). Furthermore, PLA analyses showed a time-dependent association between the supplemented recombinant TF and caveolin-1 (Figure 5). These findings are also in agreement with the study reported by Awasthi et al. [28] who used specific antibodies to TF and caveolin-1 to demonstrate the co-localisation of cell-surface TF within the caveolae. We previously reported that stimulation of HDBEC with exogenous TF resulted in exposure of fVII/fVIIa protein on the cell surface [15]. However, the location of the cellular source of fVII/fVIIa was not determined. Since factor VII may also be sequestered within caveolae, this would account for the rapid exposure of fVII/fVIIa following cellular activation, rather than either de novo expression of fVII, or the endo-vesicular transport of the fVII from the golgi apparatus. Analysis of the HDBEC using PLA indicated the presence of fVII/fVIIa within caveolae in resting HDBEC (Figure 6). Furthermore, the association between fVII/fVIIa and caveolin-1 was significantly reduced at 10 min following the incubation of the cells with recombinant TF. Interestingly, a recovery in the association between fVIIa and caveolin-1 was observed on incubation with 4 and 2 U/mL TF but not at 0.5 U/mL. Therefore, it is likely that fVII/fVIIa is released from the caveolae following cellular contact with TF to allow the activation of PAR2 and release of MV. At lower TF concentrations, the release of MV is sufficient to dispose of the cell surface TF while, at higher concentrations, the secondary mechanism of TF clearance through sequestration by the caveolae is initiated. Furthermore, an enhancement in the association between fVIIa and caveolin-1 beyond that observed in resting cells was observed following 40 min incubation with 4 U/mL TF. This observation implies that, in addition to the reservoirs within caveolae, the resting cells contain a substantial amount of exposed but latent fVII, which, in the absence of TF, does not have any influence on the behaviour of the cell. Following the exposure of the cells to TF, the interaction with the exposed fVII/fVIIa initiates a mechanism of self-regulation that is limited by the production of MV and is therefore exhaustible. Consequently, any remaining excess TF is cleared from the cell surface by the caveolae-dependent endocytosis.

However, the mechanisms involved in the dissipation of TF are not unconnected and recent evidence suggests links between caveolae and formation of microvesicle. The participation of caveolin-1 in the formation of MV extends beyond that of endocytosis and includes both cargo sorting and the processes involved in MV formation [37]. Additionally, the presence of caveolin-1 in MV have been reported [38], which appears to increase following cellular stimulation [38,39]. However, it has also been reported that PAR2 is not present in endothelial caveolae, does not interact with caveolin-1, and cholesterol depletion of endothelial cells does not affect PAR2-mediated procoagulant activity [40]. In relation to disease conditions, caveolae-dependent endocytosis has been shown to have tumour-promoting properties [41] by altering the rate of cell proliferation, migration, invasion and metastasis in cells [42,43,44,45,46], and is associated with the pro-inflammatory function of MV [47]. In contrast, caveolin-1 has also been shown to regulate the uptake of exosomes by cells [48]. Therefore, these regulations of uptake of various cargos are in turn likely to influence changes in cellular behaviour. Moreover, the transfer of proteins such as TF and PAR2 can act as a transposition of cellular properties including the procoagulant potential and the PAR2 signalling on recipient cells. To confirm the vital role of the caveolae-mediated TF clearance from the cell surface, and the detrimental consequence of retained active TF on the cell surface, the formation of caveolae in HDBEC was disrupted. Cholesterol depletion using MβCD prior to the addition of recombinant TF resulted in a significant reduction in cell numbers compared to TF alone (Figure 7). This result agrees with previous studies [30], which report that the removal of cholesterol from the cell membrane enhances the procoagulant activity of TF and therefore suggests that caveolae may act as storage for non-active TF. Our previous studies indicate that the ability of cells to moderate the level of cell-surface TF activity is a key in prevention of apoptosis [12,13,14,15,16]. This is in agreement with the hypothesis that the activation of PAR2 promotes the release of TF within MV. Furthermore, the anti-apoptotic mechanism arising from PAR2 activation may be suppressed on overexpression of caveolin-1 [49]. This implies that, despite the links between the two reported mechanisms, the signalling outcomes may be significantly dissimilar and provide a reason for the MV release as the preferred means of dissipating excess TF. In addition, it has been demonstrated that Src activation following complex formation between TF-fVIIa and β1-integrin is able to indirectly transactivate insulin growth factor-receptor 1 (IGF-R1), by suppressing the ability of caveolin-1 to block IGF-1R function [29]. However, since this mechanism was not observed following PAR2 activation, this may therefore represent the alternative and independent mechanism of TF processing.

## 5. Conclusions

In conclusion, the regulation of the amount of active TF on the cell surface is mediated through two identified mechanisms. The incorporation and release of TF within MV as a permanent means of disposing excess TF is dependent on binding to fVIIa and PAR2 activation. The alternative mechanism clears additional unreleased TF through caveolae-mediated endocytosis and is also dependent on TF-fVIIa complex formation. These mechanisms work in concert to regulate the amount of active TF on the cell surface and the signalling arising from the complexes formed.

## Data Availability

Not applicable.

## Supplementary Materials

The following are available online at https://www.mdpi.com/article/10.3390/cancers13153718/s1, Figure S1: Analysis of the assocaition of TF, fVIIa and PAR2 in BxPC-3 and 786-O cell lines using the proximity ligation assay, Figure S2: Analysis of the association of TF and PAR2 in the presence and absence of fVIIa, Figure S3: Optimisation of the labelling of HCAEC membrane using NBD-cholesterol, Figure S4: Uncropped original western blot.

## Abbreviations

TF	Tissue factor
fVII/X	Factor VII/X
fVIIa/Xa	Activated factor VII/Xa
HDBEC	Human dermal blood endothelial cells
PAR2	Protease activated receptor 2
MβCD	Methy-β-cyclodextrin
MV	Microvesicles
TF-KO cells	Tissue factor-knockout cells
