# Supplementary material for: Factor VIIa Regulates the Level of Cell-Surface Tissue Factor through Separate but Cooperative Mechanisms
Source: Cancers (Basel). 2021 Jul 23;13(15):3718. doi: 10.3390/cancers13153718 (PMC8345218; doi:10.3390/cancers13153718)
Supplement: Supplementary file 1 [file cancers-13-03718-s001.zip › cancers-1280945-supplementary.pdf]

cancers

Supplementary Material: Factor VIIa Regulates the Level of Cell-Surface Tissue Factor through Separate but Cooperative Mechanisms

Yahya Madkhali, Araci MR Rondon, Sophie Featherby, Anthony Maraveyas, John Greenman and Camille Ettelaie

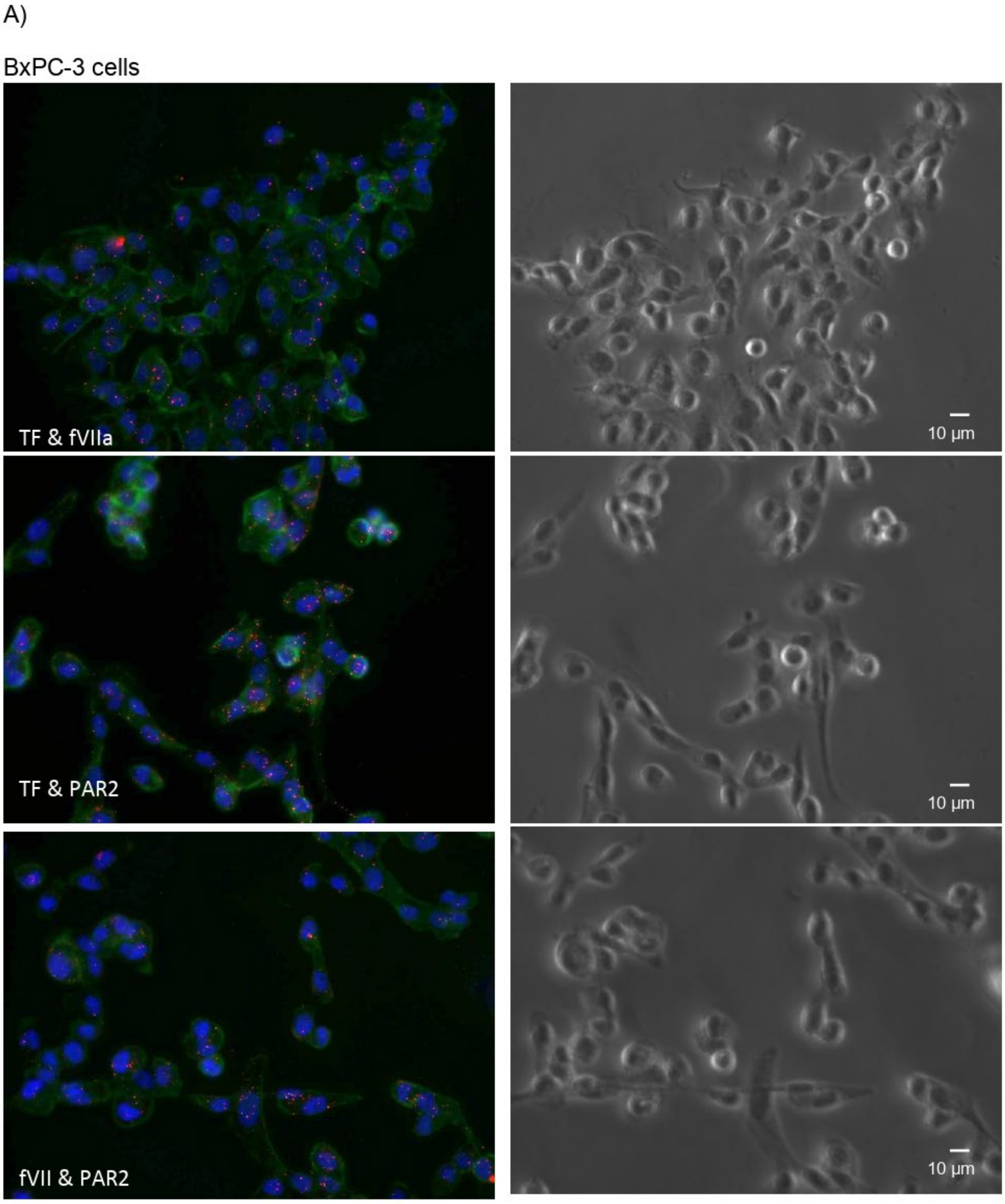

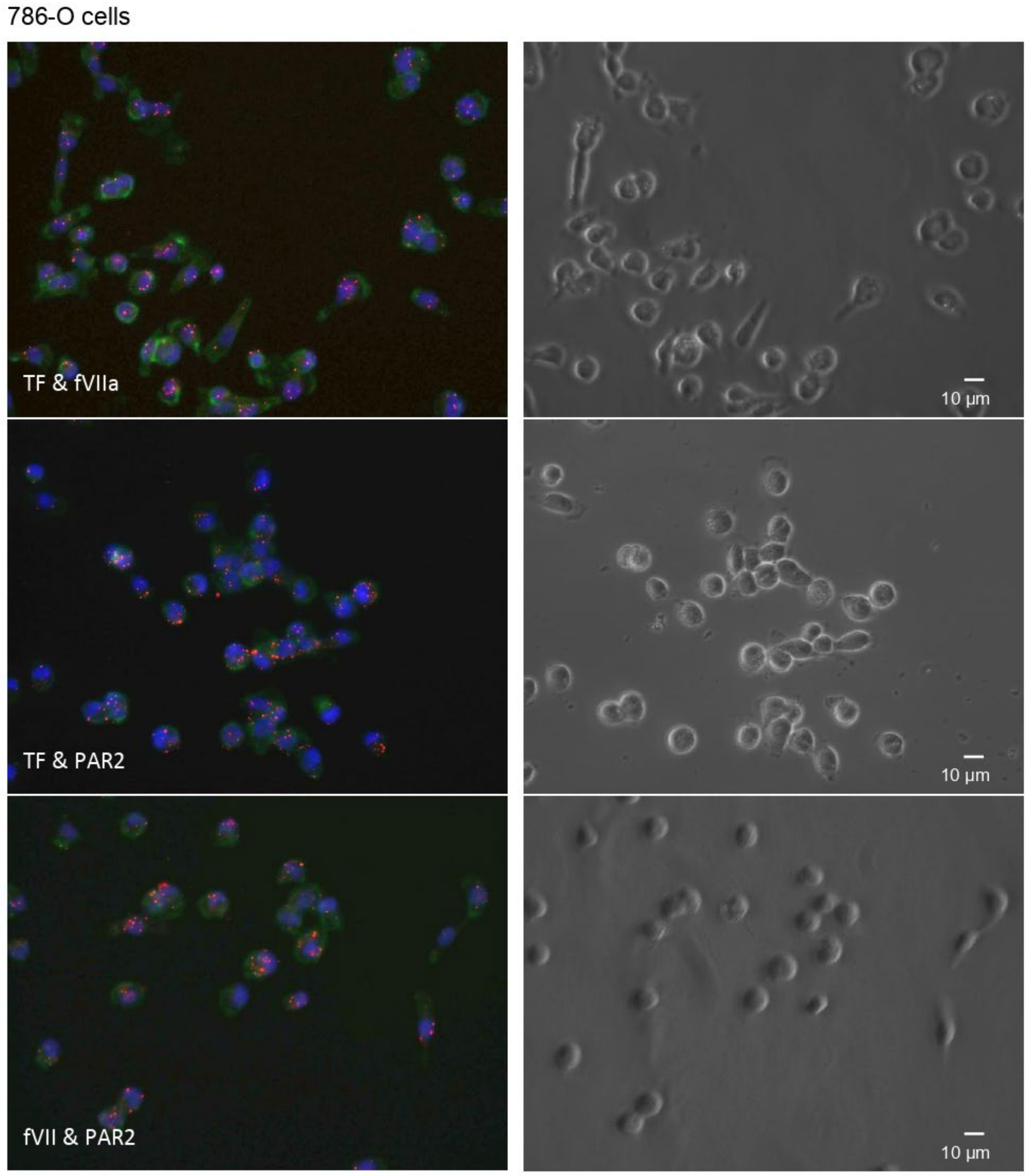
786-O cells
TF & fVIIa
10 µm
TF & PAR2
10 µm
fVII & PAR2
10 µm

B)

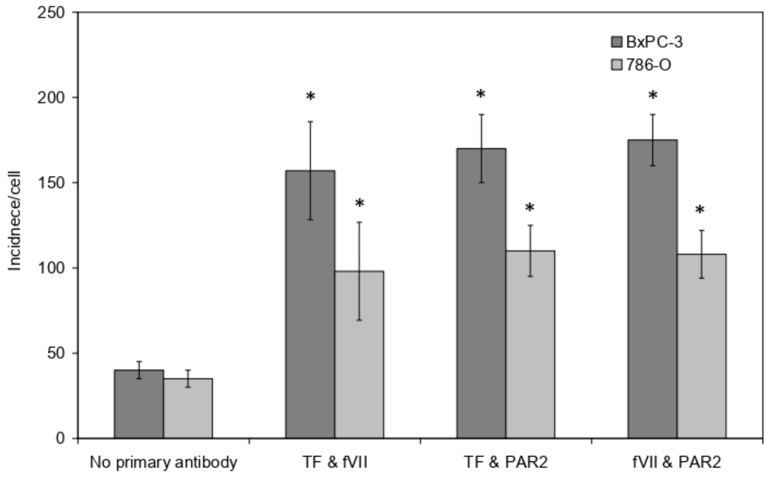
250
200
150
100
50
0
Incidnece/cell
BxPC-3
786-O
*
No primary antibody
TF & fVII
TF & PAR2
fVII & PAR2

Figure S1. Analysis of the assocaition of TF, fVIIa and PAR2 in BxPC-3 and 786-O cell lines using the proximity ligation assay. BxPC-3 and 786-O cells (10^3) were seeded out into 35 mm-glass based μ-dishes overnight. Cells were then fixed with 4% (v/v) paraformaldehyde for 15 min, washed three times with PBS for 5 min and blocked with Duolink blocking buffer for 1 h. The cells were then incubated overnight at 4°C with combinations of antibodies as follows. The cells were probed with a mouse anti-fVII antibody (321621; 10 μg/ml) together with a rabbit anti-TF antibody (FL295; 5 μg/ml) to examine the interaction between fVII and TF. Another set of cells were probed with a rabbit anti-TF antibody (FL295; 5 μg/ml) and a mouse anti-PAR2 antibody (SAM11; 20 μg/ml) to examine the association of TF and PAR2 proteins. Finally, to test the proximity between fVII and PAR2, the cells were probed with a polyclonal rabbit anti-fVII antibody (10 μg/ml) together with mouse anti-PAR2 antibody (SAM11). (**A**) The dishes were washed three times with PBS and PLA performed according to the manufacturer's instructions. The cells were stained with DAPI (2 μg/ml) and Phalloidin-FITC (2 μg/ml). Images were acquired using a Zeiss Axio Vert.A1 inverted fluorescence microscope with a ×40 magnification. (**B**) The number of red fluorescent events and nuclei were determined as incidence/cell in 10 fields of view from each assay using the ImageJ program. ($n = 4$; * = $p < 0.05$ vs the control).

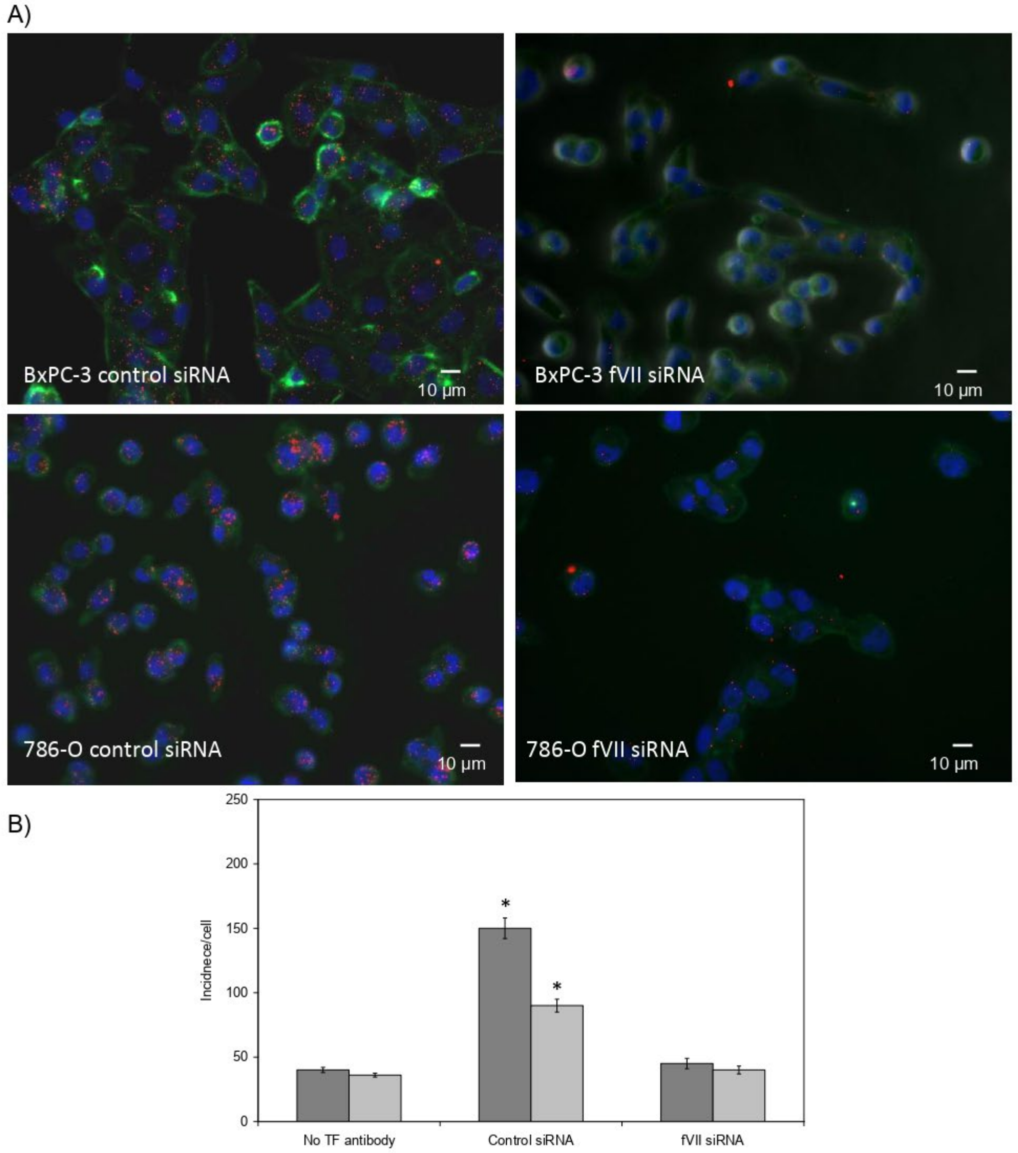

Figure S2. Analysis of the association of TF and PAR2 in the presence and absence of fVIIa. Sets of BxPC-3 and 786-O cells (10^3) were seeded out into 35 mm-glass based μ-dishes and transfected with a set of Silencer Select Pre-designed siRNA specific for the coagulation factor VII or a control siRNA (200 nM) and incubated for 48 h at 37°C. All cells were then fixed and blocked and then incubated overnight at 4°C with a rabbit anti-TF antibody (FL295; 5 μg/ml) and a mouse anti-PAR2 antibody (SAM11; 20 μg/ml). (**A**) The dishes were washed and PLA performed. The cells were stained with DAPI (2 μg/ml) and images were acquired at ×40 magnification. (**B**) The number of red fluorescent events and nuclei were determined as incidence/cell in 10 fields of view from each assay using the ImageJ program. ($n = 3$; * = $p < 0.05$ vs Sample containing control siRNA).

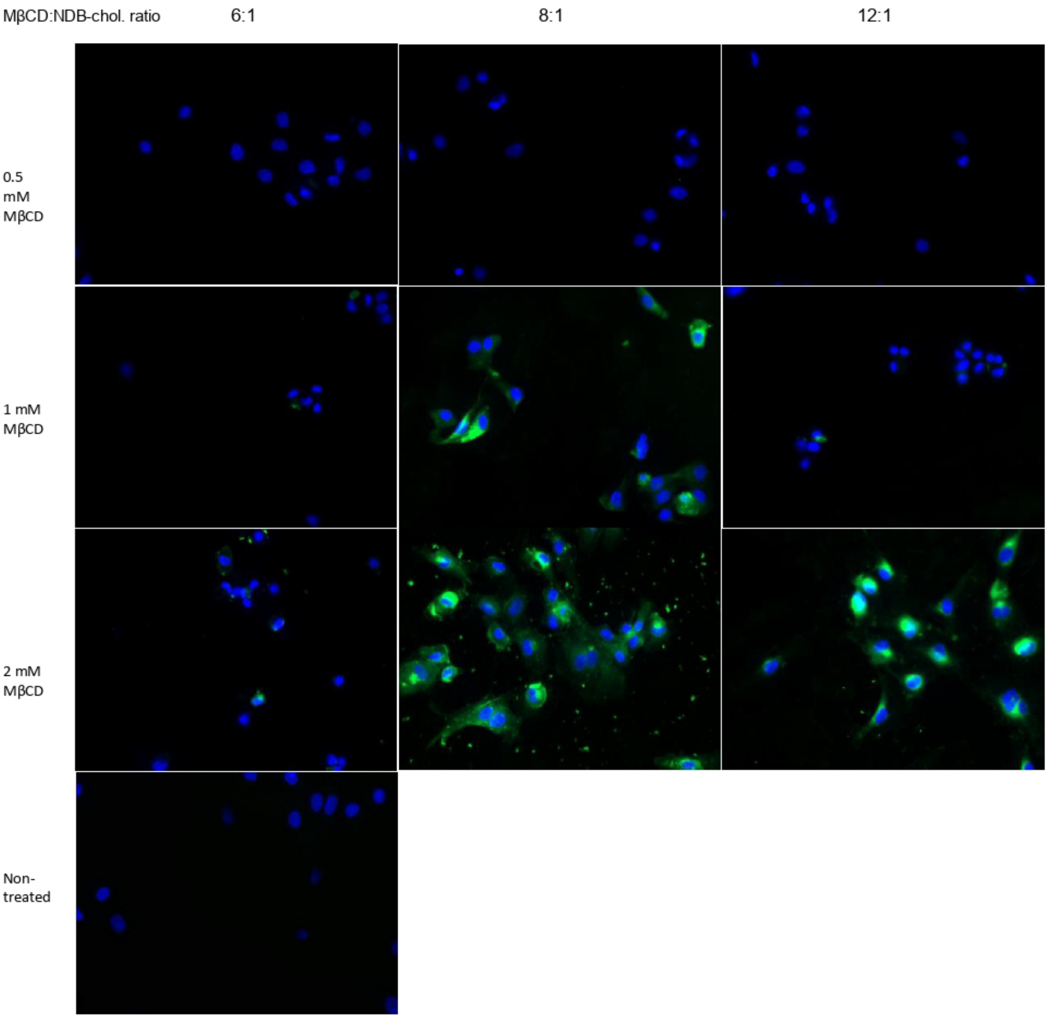

Figure S3. Optimisation of the labelling of HCAEC membrane using NBD-cholesterol. The membrane-associated cholesterol was replaced with 3-dodecanoyl-NBD cholesterol using MβCD, in order to visualise lipid rafts by fluorescence microscopy (Ext. 465 nm, Em. 535 nm). HDBEC (10^3) were seeded into 35 mm glass bottom dishes and permitted to adhere. Enrichment of the cell membranes was carried out using a range of MβCD (0.5-2 mM) and any influence on cell viability determined separately. Labelling was then carried out with combinations of MβCD:NDB-cholesterol complex at molar ratios of 6:1, 8:1 and 12:1, by incubation for 1 h at 37°C. The cells were finally washed twice with PBS and visualized using a fluorescence microscope with a ×40 magnification. Key: Green = NBD cholesterol; Blue = DAPI.

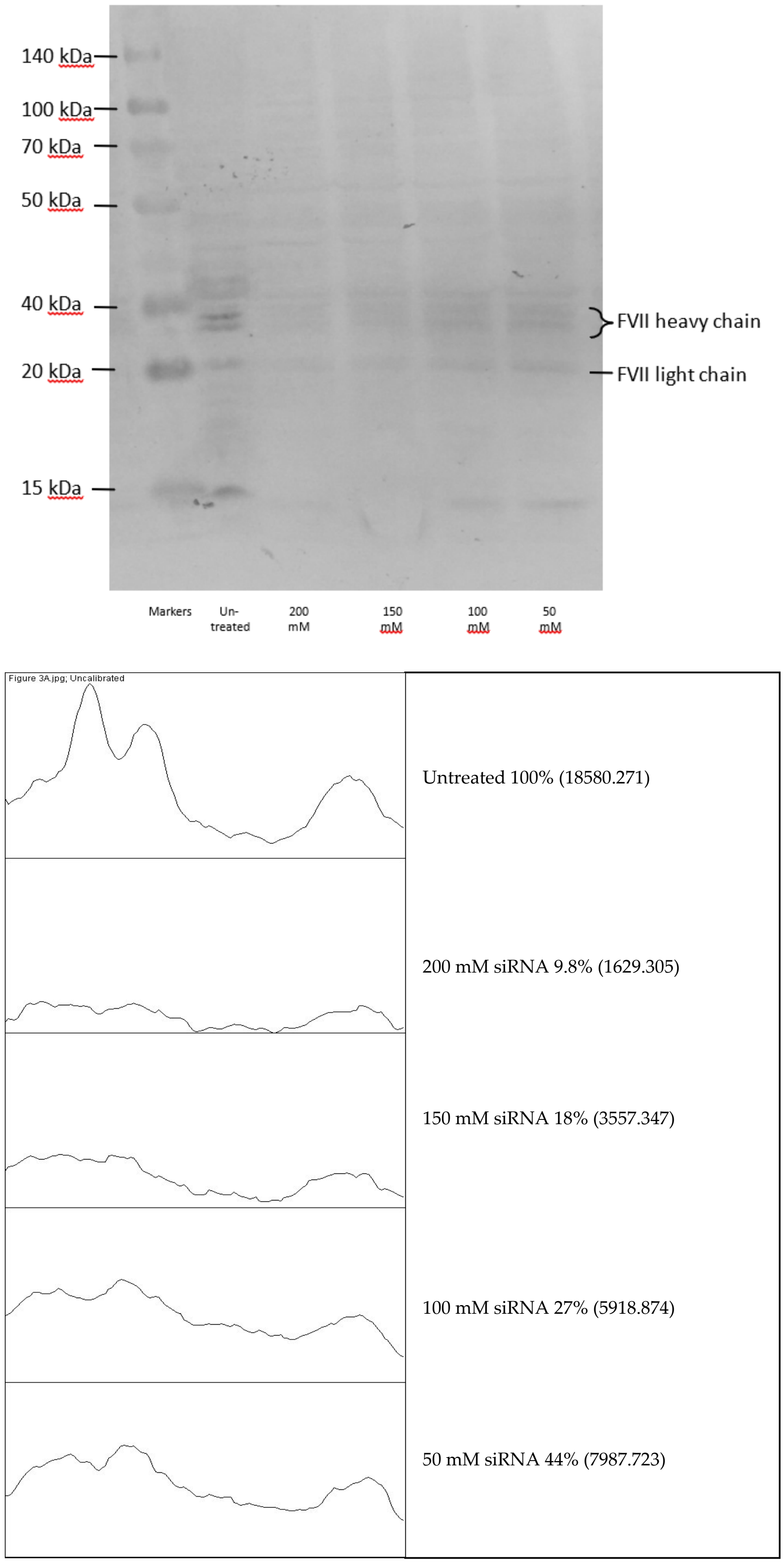

Figure S4. Uncropped original western blot.
